# Inhibition of REST Suppresses Proliferation and Migration in Glioblastoma Cells

**DOI:** 10.3390/ijms17050664

**Published:** 2016-05-03

**Authors:** Dianbao Zhang, Ying Li, Rui Wang, Yunna Li, Ping Shi, Zhoumi Kan, Xining Pang

**Affiliations:** 1 Department of Stem Cells and Regenerative Medicine, Shenyang Key Laboratory for Stem Cells and Regenerative Medicine, Key Laboratory of Cell Biology, Ministry of Public Health, and Key Laboratory of Medical Cell Biology, Ministry of Education, China Medical University, Shenyang 110122, China; zhangdianbao@gmail.com (D.Z.); liy9012@126.com (Y.L.); wangruicmu@163.com (R.W.); 2 Pharmaceutical Preparation Section, Shenyang Children’s Hospital, Shenyang 110032, China; yunnali@sina.cn; 3 Department of General Practice, the First Affiliated Hospital of China Medical University, Shenyang 110015, China; 13998309428@163.com; 4 Department of Pharmacy, the First Affiliated Hospital of China Medical University, Shenyang 110015, China; 5 Science Experiment Center, China Medical University, Shenyang 110122, China

**Keywords:** glioblastoma, REST, NRSF, proliferation, migration

## Abstract

Glioblastoma (GBM) is the most common primary brain tumor, with poor prognosis and a lack of effective therapeutic options. The aberrant expression of transcription factor REST (repressor element 1-silencing transcription factor) had been reported in different kinds of tumors. However, the function of REST and its mechanisms in GBM remain elusive. Here, REST expression was inhibited by siRNA silencing in U-87 and U-251 GBM cells. Then CCK-8 assay showed significantly decreased cell proliferation, and the inhibition of migration was verified by scratch wound healing assay and transwell assay. Using cell cycle analysis and Annexin V/PI straining assay, G1 phase cell cycle arrest was found to be a reason for the suppression of cell proliferation and migration upon REST silencing, while apoptosis was not affected by REST silencing. Further, the detection of REST-downstream genes involved in cytostasis and migration inhibition demonstrated that CCND1 and CCNE1 were reduced; CDK5R1, BBC3, EGR1, SLC25A4, PDCD7, MAPK11, MAPK12, FADD and DAXX were enhanced, among which BBC3 and DAXX were direct targets of REST, as verified by ChIP (chromatin immunoprecipitation) and Western blotting. These data suggested that REST is a master regulator that maintains GBM cells proliferation and migration, partly through regulating cell cycle by repressing downstream genes, which might represent a potential target for GBM therapy.

## 1. Introduction

Glioblastoma (GBM, WHO grade IV), the most common and malignant form of primary brain tumor, shows aggressive cancer cell proliferation and migration. Despite of improvements in surgery, chemotherapy and radiotherapy, GBM remains a cancer type with dire prognosis [1]. Due to poor responses to conventional therapies, the median survival time of GBM patients is approximately 15 months, and only 3%–5% of the patients survive more than three years [2,3]. Thus, the identification and validation of key regulators governing GBM progression is urgently needed for the development of novel treatment strategies for GBM.

The REST (repressor element 1-silencing transcription factor), also known as NRSF (neural restrictive silencing factor) and XBR (X2 box repressor), is a krüppel family zinc-finger transcription factor that binds to a well-specified DNA motif called RE1 (repressor element 1; also named NRSE, neural restrictive silencing element) within regulatory regions of genes to repress transcription by recruiting specific co-repressors multicomplexes [4,5]. REST targets include a large number of genes and accumulated studies revealed that REST plays diverse roles in multiple cellular processes [6], it was originally discovered to repress neuron-specific genes in non-neuronal tissues and neural progenitors, and it was implicated as a tumor suppressor in breast cancer, colorectal cancer and small cell lung cancer, and as an oncogene in neuroblastomas, medulloblastomas and pheochromocytomas [7,8]. In GBM, previous studies showed enhanced REST protein levels in patient-derived specimens and tumorigenic-competent GBM cells [9], and inhibition of REST in GBM isolated cancer stem cells and subsequent implantation of these cells into mice resulted in tumors that were significantly less invasive, highly apoptotic and slower growing [10,11,12], partly through regulating tumor suppressor microRNA miR-124a and its downstream targets [13]. However, the detailed function of REST and its mechanisms in GBM are not very clear.

In the present study, we aimed to investigate the crucial roles of REST in proliferation and migration of human GBM cells, as well as the underlying mechanisms, which might benefit the future therapy for malignant gliomas.

## 2. Results

### 2.1. Repressor Element 1-Silencing Transcription Factor (REST) Knockdown by siRNA

**Figure 1:**
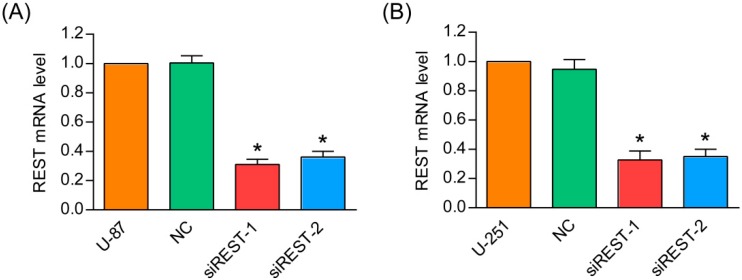

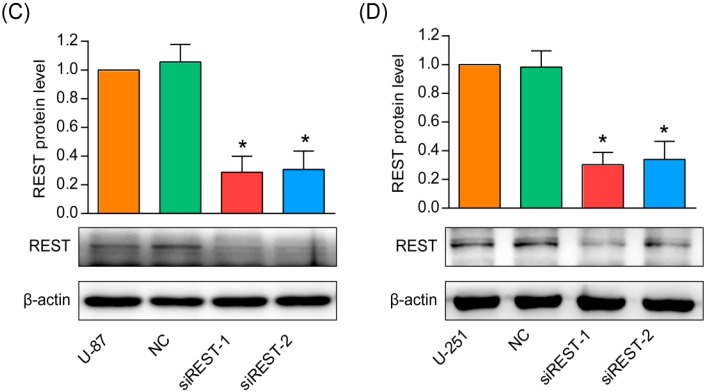
siRNA-mediated down-regulation of repressor element 1-silencing transcription factor (REST) in glioblastoma (GBM) cells. After U-87 and U-251 cells were transfected with siREST-1, siREST-2 or NC (scramble siRNA as negative control) for 48 h, the mRNA and protein levels of REST were determined by real-time PCR and Western blotting, respectively. (**A**) mRNA levels of REST in transfected U-87 cells; (**B**) mRNA levels of REST in transfected U-251 cells; (**C**) protein levels of REST in transfected U-87 cells; (**D**) protein levels of REST in transfected U-251 cells. * *p* < 0.05 compared with NC group.

Previous studies have shown that REST is overexpressed in GBM cells and tissues [10,11]. To investigate the functional role of REST in GBM, siRNA was used to knockdown REST expression in U-87 and U-251 cells. Two different siRNAs (siREST-1 and siREST-2) designed against different regions of REST were tested for their efficiency in knocking down. As shown in Figure 1A,B, REST mRNA level was significantly reduced to about 30% in U-87 and U-251 cells at 48 h after transfection with siREST-1 or siREST-2, compared with NC (scramble siRNA as negative control). Further, the silencing efficiencies of the two siRNAs against REST were confirmed at the protein level, and changes observed by Western blotting were consistent with the results in real-time PCR (Figure 1C,D). These results indicated that the specific siRNA targeting REST/NRSF was able to effectively knockdown endogenous REST at both mRNA and protein levels in U-87 and U-251 GBM cells.

### 2.2. REST Knockdown Reduced Cell Proliferation

**Figure 2:**
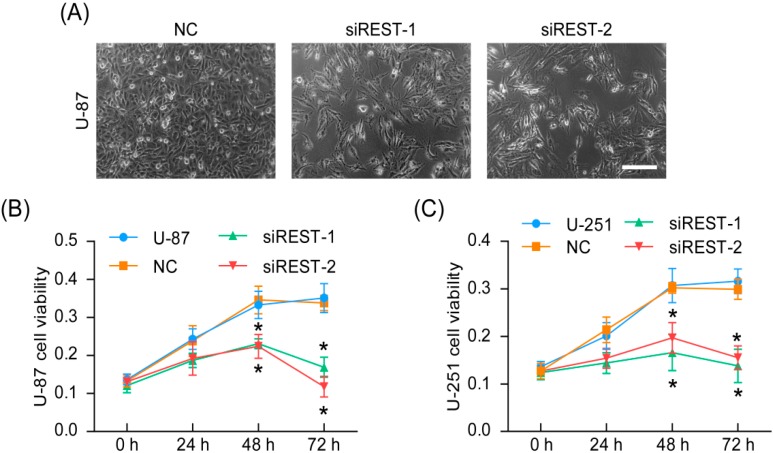
Cell proliferation was inhibited by REST silencing. (**A**) Representative phase-contrast micrographs of transfected U-87 cells; (**B**) cell viability of transfected U-87 cells; (**C**) cell viability of transfected U-251 cells. * *p* < 0.05 compared with NC group. Scale bar indicates 200 nm.

The representative phase-contrast micrographs had revealed that U-87 cells with REST knockdown had fewer cells than those of negative control at 72 h after transfection (Figure 2A). Further, the cell viability was quantified using CCK-8 assay and as shown in Figure 2B,C, the cell viabilities of U-87 and U-251 cells were significantly decreased at 48 and 72 h after transfection with siREST-1 or siREST-2 in comparison with NC. Together, these results suggested that REST was critical for maintenance of GBM cells proliferation and siRNA targeting REST may serve as a potential therapy strategy.

### 2.3. G1 Phase Cell Cycle Arrest Induced by REST Silencing

**Figure 3:**
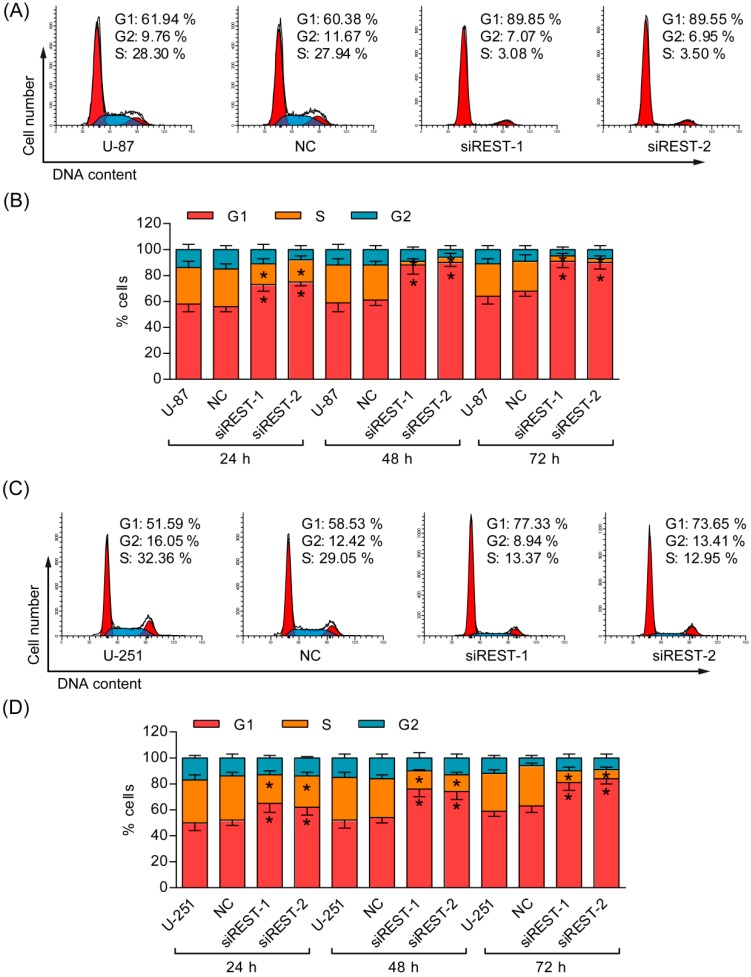
Knockdown of REST induced G1 phase arrest. (**A**) Representative cell cycle plots of U-87 cells at 48 h after REST knockdown; (**B**) The cell cycle distribution of 3 independent experiments at 24, 48 and 72 h in U-87 cells; (**C**) Representative cell cycle plots of U-251 cells at 48 h after REST knockdown; (**D**) The cell cycle distribution of 3 independent experiments at 24, 48 and 72 h in U-251 cells. * *p* < 0.05 compared with NC group.

**Figure 4:**
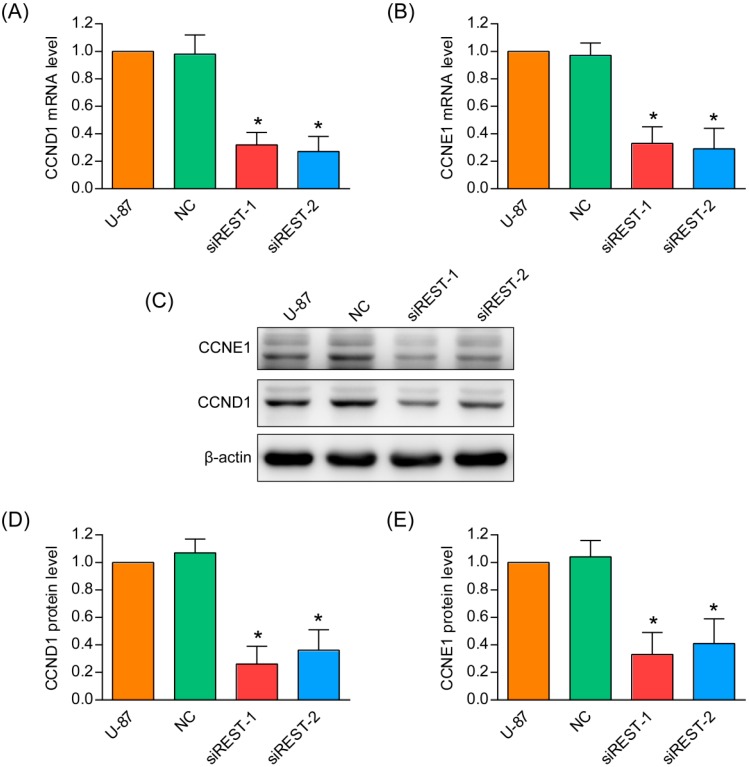
CCND1 and CCNE1 were reduced by REST knockdown. (**A**,**B**) The mRNA levels of two genes involved in G1–S cell cycle transition, CCND1 and CCNE1 were analyzed by real-time PCR; (**C**) The expression of CCND1 and CCNE1 was verified by Western blotting; (**D**,**E**) The quantifications of Western blotting were applied with Image J. * *p* < 0.05 compared with NC group.

To understand the mechanisms by which cell proliferation was affected, the percentages of cells in different phases were analyzed by flow cytometry. After transfection with siREST-1 or siREST-2, U-87 and U-251 cells accumulated at the G1 phase, with a concomitant reduction in the proportion of cells in the S phase and a small decrease of cells in the G2 phase as compared with NC-transfected and non-treated cells (Figure 3). Meanwhile, real-time PCR and Western blotting analysis in U-87 cells demonstrated that REST knockdown significantly reduced both mRNA and protein levels of CCND1 and CCNE1 (Figure 4), which have critical roles in regulating the entry of cells into S phase at the G1/S transition checkpoint. These data demonstrated that the induction of G1 phase arrest accounted for the inhibitory effects of REST/NRSF knockdown on cell proliferation.

### 2.4. REST Silencing Does Not Induce Cell Apoptosis

**Figure 5:**
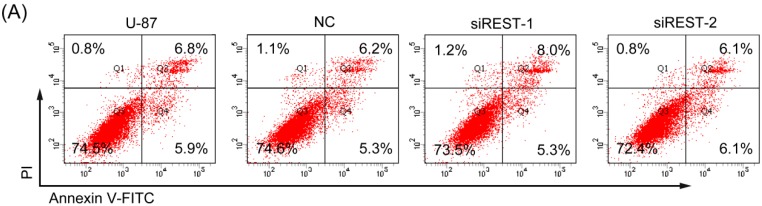

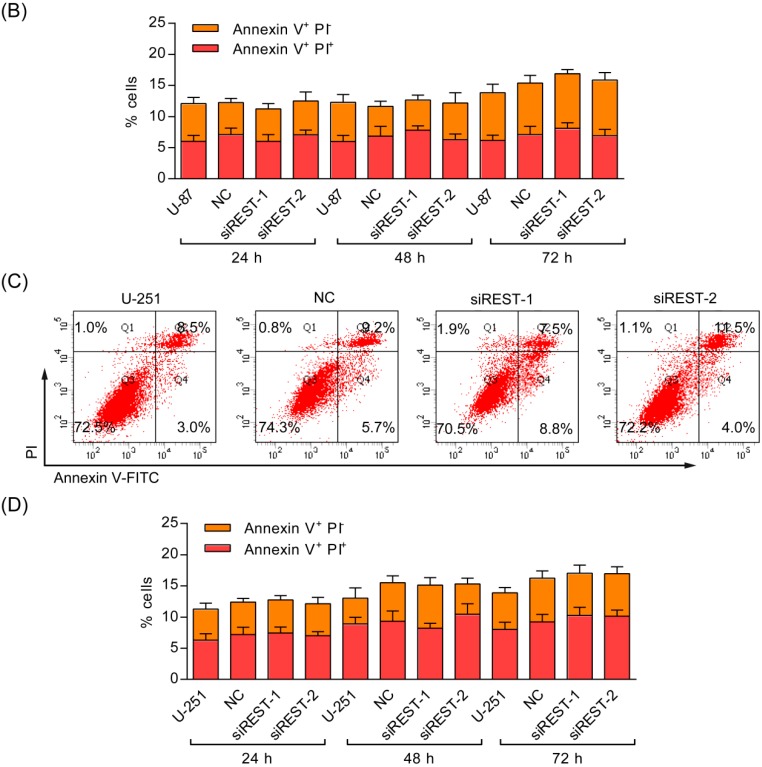
REST silencing does not induce cell apoptosis. (**A**) Representative cell apoptosis plots of U-87 cells at 48 h after REST knockdown; (**B**) The apoptosis of 3 independent experiments at 24, 48 and 72 h in U-87 cells; (**C**) Representative cell apoptosis plots of U-251 cells at 48 h after REST knockdown; (**D**) The apoptosis of 3 independent experiments at 24 h, 48 h and 72 h in U-251 cells.

Given that the cell viability was obviously reduced by REST silencing in GBM cells, Annexin V/PI staining was employed to determine whether the inhibitory effect was related to apoptosis. As shown in Figure 5, there were no significantly changes of early, as well as, late apoptosis that occurred in U-87 and U-251 cells upon REST knockdown. Additionally, in the results of cell cycle analysis, the characteristic hypodipolid peak (subG1), indicating apoptotic cells, did not appear after 48 h of transfection (Figure 3A,C), consistent with the apoptosis assay described above. Thus, apoptosis may not contribute to reduced cell viability upon REST knockdown.

### 2.5. Cell Migration Is Decreased by REST Silencing

**Figure 6:**
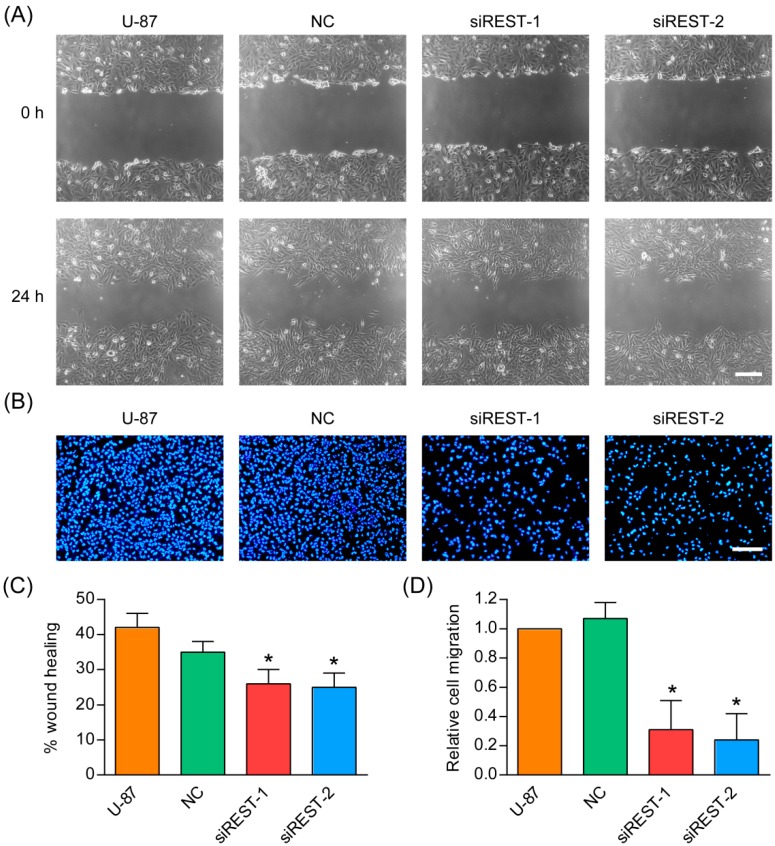
REST silencing reduced migration of U-87 cells. (**A**) *In vitro* scratch assay carried out with U-87 cells over 2 h; (**B**) Transwell migration assay was conducted with U-87 cells over 12 h; (**C**) The quantification of wound healing assay; (**D**) The quantification of transwell assay. * *p* < 0.05 compared with NC group. Scale bars in **A** and **B** indicate 200 nm.

**Figure 7:**
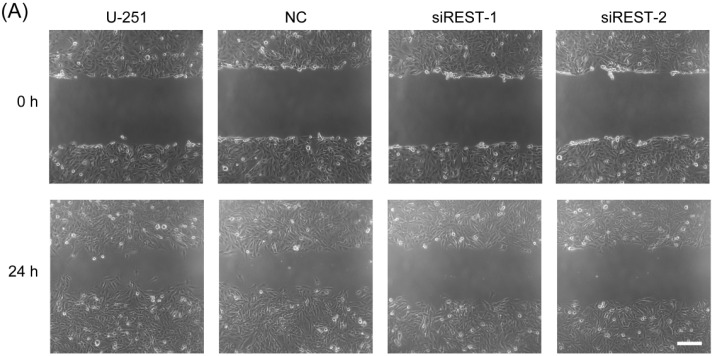

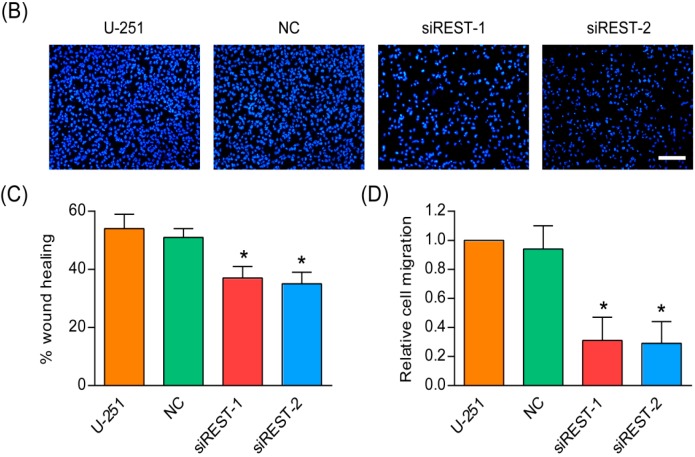
REST silencing reduced migration of U-251 cells. (**A**) *In vitro* scratch assay carried out with U-251 cells over 24 h; (**B**) Transwell migration assay was conducted with U-251 cells over 12 h; (**C**) The quantification of wound healing assay; (**D**) The quantification of transwell assay. * *p* < 0.05 compared with NC group. Scale bars in **A** and **B** indicate 200 nm.

Migration is a critical step in initial progression of cancer that facilitates metastasis. In order to study the influence of REST knockdown on the migration potential of GBM cells, transfection in U-87 and U-251 cells was performed as described, *in vitro* scratch wound healing assay and transwell migration assay were subsequently applied. As shown in the results of wound healing assay (Figure 6 and Figure 7), compared with the NC-transfected and non-treated U-87 and U-251 cells, at 24 h after scratching, REST knockdown cells were less efficient in migrating and closing wounds. Consistent with the results obtained from the wound healing assay, a transwell assay revealed that REST silencing significantly decreased migration of U-87 and U-251 cells. In summary, down-regulation of REST by siRNA silencing could inhibit the migration of GBM cells.

### 2.6. REST Knockdown Enhanced Genes Involved in Cytostasis and Migration Inhibition

**Figure 8:**
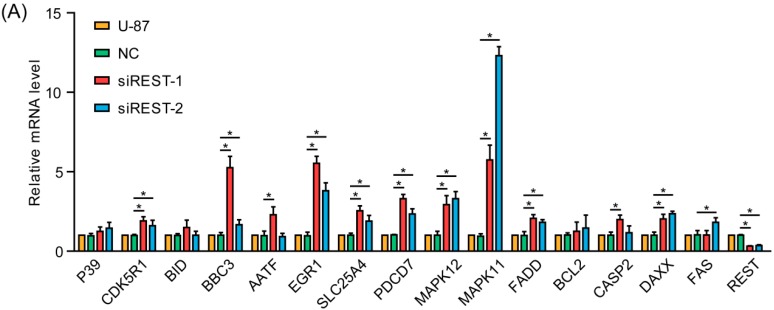

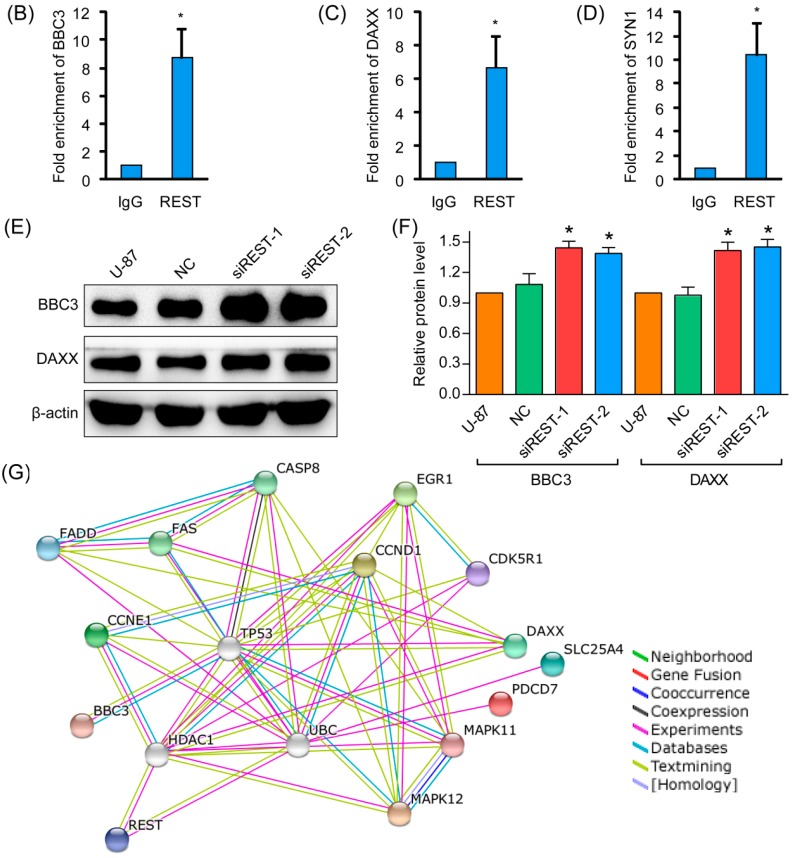
REST silencing elevated genes involved in cytostasis and migration inhibition. (**A**) The mRNA level of potential REST-regulated genes involved in cytostasis and migration inhibition were analyzed by real-time PCR, and GAPDH was used as an internal control; (**B**,**C**) ChIP (chromatin immunoprecipitation) assays were performed to pull down REST-DNA complexes from U-87 cells using anti-REST antibodies, and fold enrichment of BBC3 and DAXX was determined by real-time PCR; (**D**) SYN1 was used as a positive control; (**E**,**F**) The protein level of BBC3 and DAXX were verified by Western blotting; (**G**) The interactome was generated from the identified upregulated genes with the help of STRING database, using medium confidence (0.400) parameter, in order to identify highly-possible connections. * *p* < 0.05 compared with NC group.

To gain more insight into the consequences of REST silencing in GBM cells, the expression of potential REST-regulated genes involved in cytostasis and migration inhibition [14,15] were analyzed by real-time PCR. As shown in Figure 8A, the mRNA levels of CDK5R1, BBC3, EGR1, SLC25A4, PDCD7, MAPK11, MAPK12, FADD and DAXX were elevated after REST knockdown, GAPDH was used as an internal control. According to the high-throughput genome-wide studies [14], among the above-mentioned REST-responsive genes, BBC3 and DAXX were shown to be potential REST-binding targets. Thus, ChIP (chromatin immunoprecipitation) assays were carried out with U-87 cells to further investigate whether REST can be bound to the promoters of these genes. The results showed significant enrichment of REST on BBC3 and DAXX promoters compared with IgG by using anti-REST antibodies (Figure 8B,C), indicating transcriptional suppression of the genes. The positive control SYN1 was also significantly enriched in ChIP (Figure 8D). Further, the upregulated expression of DAXX and BBC3 were verified at the protein level (Figure 8E,F). By using the STRING database, the interactome was generated from the identified enhanced genes, at medium confidence (0.400) parameter in order to identify highly possible connections (Figure 8G). It helps to discover the proteins playing key roles in the interactome developed from the identified proteins, and the results revealed HDAC1 and UBC as media nodes linking REST and its downstream genes. The results indicated that transcriptional up-regulation of these genes may contribute to the cytostasis and migration inhibition upon REST silencing, among which BBC3 and DAXX were direct targets of REST.

## 3. Discussion

GBM is a cancer type composed of genetically diverse cells, but always overexpresses genes vital to cell cycle regulation, cell growth and proliferation, cell migration and invasion [16,17]. Previous studies in patient-derived specimens and tumorigenic-competent GBM cells showed upregulated REST expression for two- to five-fold as compared to normal [9,11], and high REST expression coincided with high levels of SOX2, which is known as a neural stem cell self-renewal regulator [10]. REST depletion mediated by lentivirus could strongly reduce self-renewal potential and induced neuronal differentiation and cell death programs in human GBM cell lines established from fresh GBM surgical specimens [11]. Additionally, it was reported that pioglitazone, through down-regulating REST mRNA level, could inhibit U-87 cell proliferation [9]. High levels of REST activity may play a central role in the tumorigenesis and development of GBM. Consistent with this possibility, we found that the proliferation and migration capacity were both reduced considerably upon REST silencing by siRNA in U-87 and U-251 GBM cells. Moreover, to avoid siRNA off-target effects [18], two different siRNAs sequences designed against different regions of REST were used. Further, the cell cycle distribution and apoptosis were detected, it was revealed that REST knockdown inhibited the progression from G1 to S phase of the cell cycle, during which two key regulators CCND1 and CCNE1 were downregulated. These data suggested that G1 phase cell cycle arrest was a main reason for the suppression of cell proliferation and migration upon REST silencing.

REST is a transcription repressor associated with gene regulation and it plays diverse roles in multiple cellular processes. REST has been implicated as a tumor suppressor in nonneural tumors and as an oncogene in neural tumors [4,8], and previous studies have uncovered that several mechanisms are involved. In medulloblastomas, REST expression could decrease cyclin-dependent kinase (CDK)NIB/p27 (a CDK inhibitor) by repressing ubiquitin specific peptidase 37 (USP37), which could form a complex with p27 to promote its deubiquitination and stabilization, and resulting in blocked cell proliferation [19]. Additionally, raised expression of REST in pheochromocytoma cell line could increase oncogenes Myc and CCND1, which might account for the proliferation advantage and the distinct morphology [20]. More recently, studies in GBM found that high NRSF expression inhibited miR-124a, thereby increasing the expression of SNAI-1, Scp1 and PTPN12, promoting cell proliferation and invasion [10,11,13]. In this study, to investigate the underlying mechanisms involved in the suppression of cell proliferation and migration upon REST silencing, we performed realtime PCR to analyze involved REST-downstream genes, and CDK5R1, BBC3, EGR1, SLC25A4, PDCD7, MAPK11, MAPK12, FADD and DAXX were found to be significantly upregulated, among which BBC3 and DAXX were found to be direct targets of REST, as verified by ChIP and Western blotting. Bioinformatic analysis of known and predicted protein–protein interactions using STRING v10 revealed that, in these proteins’ interaction network, HDAC1 and UBC (ubiquitin C) were hub nodes, linking REST and its downstream genes, indicating the potential mechanisms of histone deacetylation and protein ubiquitination in the quantity control of these genes at the transcriptional and protein levels.

GO (Gene Ontology) analysis and Pathway analysis of these REST-downstream genes showed significant enrichment for genes in the MAPK pathway and genes that are associated with cell survival, which could potentially explain the observed phenotypes in response to REST siRNA treatment. Among these genes, CCND1 and CCNE1 act as key regulators of the cell cycle, which are upregulated during G1–S transition [21]. The down-regulation of these two genes upon REST silencing accounted for the cell cycle arrest at the G1 phase in U-87 and U-251 GBM cells. Upregulated MAPK11 and MAPK12 are two of the four p38 MAPKs that play important roles in the cellular responses evoked by extracellular stimuli, and MAPK12 plays a role in repressing cell proliferation through the down-regulation of cyclin D1 in response to hypoxia in adrenal cells [22]. At the same time, CDK5R1 is a neural specific activator of CDK5, which could regulate glioblastoma cell migration and invasion [23]. It was also reported that the suppression of the transcription factor EGR1 is a common event in gliomas, resulting cell proliferation [24], so the upregulation could contribute to the inhibition effects in GMB cells upon REST silencing. Additionally, the genes DAXX, BBC3, PDCD7, FADD and SLC25A4 all encode anti-proliferation proteins; a large number of studies have shown that the overexpression of these genes could promote cell apoptosis or necrosis. It was suggested that, partly through upregulating these genes, REST knockdown inhibited cell proliferation and migration in GBM cells.

**Figure 9:**
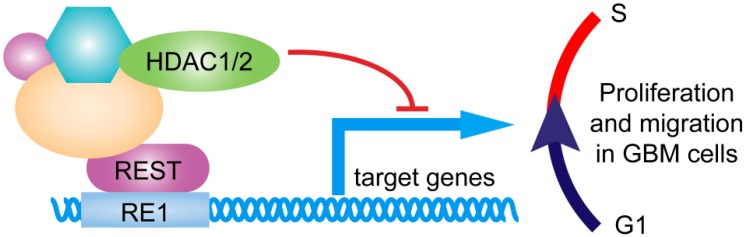
REST was a key regulator of GBM cells proliferation and migration. REST binds to RE1 motif and recruits cofactors to repress downstream genes, triggering cell cycle G1–S transition to maintain proliferation and migration in GBM cells.

Epigenetic modification was the main mechanism for REST to repress its target genes. REST could bind to RE1 motif and form co-repressor complexes by recruiting cofactors, such as Sin3, CoREST, Polycomb Repressive Complexes, and various histone deacetylases (HDACs) [4]. Where HDAC 1 and 2 were recruited by mSin3A for histone deacetylation [25], site-specific histone methyltransferase G9a could promote H3K9me2 dimethylation [26], site-specific histone demethylation LSD1 enzyme was able to remove methyl group of H3K4me1 and HSK4me2 [26,27], CpG binding protein MeCP2 can read histidine epigenetic markers and DNA methylation [28]. Histone acetylation levels are dynamic markers for gene expression, while methylation status of histones and DNA related to long-term gene expression stability [29]. In this study, the upregulation of REST downstream genes, especially for BBC3 and DAXX, occurred after a relatively short period of REST silencing, indicating that BBC3 and DAXX may be suppressed by recruiting HDAC1/2, coinciding with the bioinformatics analysis above (Figure 9).

In summary, various mechanisms have been identified to support the oncogenic role of REST in human GBM. REST acted as a master regulator that maintains GBM cells proliferation and migration, partly through regulating cell cycle by repressing downstream genes. Thus, REST may serve as a potential therapeutic target for GBM tumors.

## 4. Materials and Methods

### 4.1. Cell Culture

Human GBM cell line U-87 and U-251 was purchased from Cell Bank of Chinese Academy of Sciences. The cells were maintained in Dulbecco’s Modified Eagles Medium (DMEM) (HyClone, Beijing, China) supplemented with 10% fetal bovine serum (FBS) (HyClone) and 1% Penicillin streptomycin (Gibco, Carlsbad, CA, USA) at 37 °C in a humidified atmosphere containing 5% CO_2_.

### 4.2. siRNA Transfection

GBM cells were seeded in culture plates and maintained for 12 h before transfection. Lipofectamine RNAiMAX Transfection Reagent (Invitrogen, Carlsbad, CA, USA) was used for siRNA delivery with Opti-MEM I Reduced Serum Medium (Gibco) according to the manufacturer’s instruction, and the culture medium was replaced at 6 h after transfection [30]. 100 and 5 pmol siRNA were used for each well of 6-well and 96-well plates separately. For human REST/NRSF silencing, 2 siRNA sequences were used and the sequence were as follows: siREST-1: 5′-GCUGCGGCUACAAUACUAATT-3′; siREST-2: 5′-GCUUAUUAUGCUGGCAAAU-3′ [31]. Additionally, the sequence of NC was 5′-UCCGAACGUGUCACGUTT-3′. All the siRNA duplexes were chemically synthesized by GenePharma (Suzhou, China).

### 4.3. In Vitro Scratch Assay

The cells were transfected and maintained to 90%–100% confluence in 6-well culture plates. Then the cell monolayers were scratched using a sterile 200-μL pipette tip and then washed with PBS to remove cellular debris. The wounded monolayers were incubated and images were taken at time 0 and 24 h post-wounding under an inverted microscope (Axiovert 40 CFL, Carl Zeiss, Oberkochen, Germany). The wound closure was analyzed using TScratch software (CSElab, Zurich, Switzerland).

### 4.4. Transwell Migration Assay

The migration ability of GBM cells was determined in Boyden Chamber. 24 h after transfection, the cells were seeded into 8-μm Transwells (6.5 mm diameter, Corning, Tewksbury, MA, USA) at 5 × 10^4^ cells per well with serum-free culture medium. And medium containing 10% FBS was added into the lower chamber and served as the chemoattractant. After incubation for 12 h, the cells remaining on the upper surface of the filter were removed by gently wiping with a cotton swab. The cells migrated through the filter were fixed with methanol, stained with 4′,6-diamidino-2-phenylindole (DAPI) and visualized by inverted fluorescence microscope (Olympus IX51, Tokyo, Japan). The graphs were analyzed using ImagePro Plus 6.0 software (Media Cybernetics, Rockville, MD, USA).

### 4.5. Cell Proliferation Assay

The cell proliferation was assessed by Cell Counting Kit-8 (CCK-8, Dojindo, Kumamoto, Japan). Cells were seeded in 96-well plates at 4000 cells per well and siRNA transfection was carried out as described. After 10 μL CCK-8 reagent was added, the cells were continuously incubated for 2 h. The spectrophotometric absorbance of the samples was measured with a microplate reader iMARK (Bio-Rad, Hercules, CA, USA) at 450 nm with a reference wavelength of 630 nm.

### 4.6. Cell Cycle Analysis

The cells were collected and fixed with ice-cold 70% ethanol overnight at 4 °C, Cell Cycle and Apoptosis Analysis Kit (Beyotime, Nantong, China) were used for cell cycle analysis. The fixed cells were stained with 0.5 mL of propidium iodide (PI) staining buffer (contains 200 mg/mL RNase A and 50 μg/mL PI) at 37 °C for 30 min in the dark. PI-stained cells were analyzed by flow cytometry (BD Biosciences, Franklin Lakes, NJ, USA).

### 4.7. Cell Apoptosis Assay

Cell apoptosis analysis was carried out by Annexin V-FITC Apoptosis Detection Kit (Sigma, Saint Louis, MO, USA) following manufacturer’s instruction. In brief, cells were harvested by trypsinization and resuspended in binding buffer. Annexin V-FITC and PI were added to 500 μL of cell suspension and incubated for 10 min in the dark at room temperature. The cells were immediately analyzed by flow cytometry (BD Biosciences).

### 4.8. Quantitative Real-Time PCR

Total RNA was isolated using TRIzol Reagent (Ambion, Carlsbad, CA, USA) and quantified by NanoDrop 2000C spectrophotometer (Thermo, Wilmington, DE, USA). cDNA was synthesized using PrimeScript RT reagent Kit with gDNA Eraser (Takara, Dalian, China) and quantitative real-time PCR was carried out using SYBR Select Master Mix (Invitrogen, Carlsbad, CA, USA) in ABI 7500 Real-Time PCR System. The relative gene expression was calculated using the ΔΔ*C*_t_ method, and GAPDH was used as an internal control. All the primers were chemically synthesized by AuGCT Biotech (Beijing, China) and the sequences are listed in Table 1 [32].

### 4.9. Western Blotting Analysis

Cell lysates were prepared in ice-cold RIPA lysis buffer (Beyotime, Haimen, China) and the protein concentrations of the samples were determined using a BCA protein assay kit (Dingguo, Beijing, China). Equal amounts of proteins were separated by SDS-PAGE electrophoresis, transblotted on PVDF membrane and probed with rabbit anti-REST polyclonal antibody (1:1000, Millipore, Temecula, CA, USA), rabbit anti-CCND1 antibody (1:1000, Abcam, Cambridge, MA, USA), rabbit anti-CCNE1 antibody (1:1000, Proteintech, Wuhan, China), rabbit anti-BBC3 polyclonal antibody (1:1000, Affinity Biosciences, Zhenjiang, China), rabbit anti-DAXX polyclonal antibody (1:1000, Affinity Biosciences, Zhenjiang, China) and mouse anti-β-actin antibody (1:5000, Proteintech, Rosemont, IL, USA). After incubation with HRP-conjugated secondary antibodies (1:5000, KangChen, Shanghai, China), protein bands were visualized using Amersham ECL Prime Western Blotting Detection Reagent (GE Healthcare, Princeton, NJ, USA) on Tanon-5200 chemiluminescence detection system. The bands were analyzed by using Image J software (NIH, Bethesda, MD, USA).

### 4.10. Chromatin Immunoprecipitation (ChIP)

ChIP was performed by using ChIP-IT Express Enzymatic Kit (Active motif, Carlsbad, CA, USA) according to modified manufacturer’s instructions. Briefly, U-87 MG cells cultured in 15-cm plates were fixed and collected by centrifugation. The pellet was resuspended and lysed in ice-cold Lysis Buffer on ice for 30 min. Chromatin was sheared by enzymatic digestion for 10 min at 37 °C. DNA concentration and shearing efficiency was determined by DNA cleanup and agrose gel electrophoresis. Subsequently, 3 μg ChIPAb+ rabbit anti-REST polyclonal antibody (Millipore) and Normal Rabbit IgG (negative control, Millipore) was used for immunoprecipitation with protein G Magnetic beads. The bound chromatin was then eluted from the beads and incubated in Reverse Cross-linking Buffer for 2.5 h at 65 °C, followed by Proteinase K treatment. DNA cleanup procedure was carried out using E.Z.N.A. Cycle-Pure kit (Omega Bio-Tech, Norcross, GA, USA). For quantitative ChIP, real-time PCR was performed, as described above, with the listed primers (Table 1). SYN1 was applied as a positive control.

### 4.11. Protein Interactions Analysis by Search Tool for the Retrieval of Interacting Genes/Proteins (STRING)

The interactomes of genes/proteins were analyzed by using Search Tool for the Retrieval of Interacting Genes/Proteins (STRING) database [15], followed by biological process (GO) analysis and KEGG (Kyoto Encyclopedia of Genes and Genomes) pathways analysis. The protein-protein interaction network of the identified proteins was generated at a medium confidence value (0.400) to identify highly possible connections.

### 4.12. Statistical Analysis

Data were expressed as the mean ± SD from at least three independent experiments. Statistical analysis between two samples was performed using Student’s *t*-test. And statistical comparisons of more than two groups were performed using one-way analysis of variance (ANOVA). All statistical analyses were performed using SPSS 16.0 software (SPSS Inc., Chicago, DE, USA). *p* < 0.05 was considered statistically significant.

## 5. Conclusions

In the present study, our results established REST as a key regulator of both proliferation and migration in GBM cells. REST silencing by siRNAs led to a significant reduction of cell proliferation and migration capacities in U-87 and U-251 GBM cells, during which G1 cell cycle arrest accounted for the inhibition effect, while cell apoptosis was not affected by REST silencing. The data also shed light on some downstream genes of REST that control GBM proliferation and migration, including CDK5R1, BBC3, EGR1, SLC25A4, PDCD7, MAPK11, MAPK12, FADD and DAXX, among which BBC3 and DAXX were direct targets of REST. REST knockdown may reduce the bound REST on the promoters of these genes, triggering their transcription to suppress GBM progression. These might represent potential targets for GBM therapy.

## Figures and Tables

**Table 1:** Primer sequences for real-time PCR.

Gene	Primer Sequence	Product Length (bp)
*P39*	forward: 5′-CCTTCATTACGCCTGCAAA-3′	144
	reverse: 5′-TCTCGTTGCCCATGTAGGA-3′	
*CDK5R1*	forward: 5′-CAAACCAGGAGCATTTTGTGT-3′	91
	reverse: 5′-ATTCCTGTGGCTTGTTCTGTG-3′	
*BID*	forward: 5′-AGTGGGAGGGCTACGATGAG-3′	155
	reverse: 5′-GATGCTACGGTCCATGCTGT-3′	
*BBC3*	forward: 5′-CCCGTGAAGAGCAAATGAG-3′	153
	reverse: 5′-ACCCCCTGATGAAGGTGAG-3′	
*AATF*	forward: 5′-GCCAGGATCGTCTGATGAGG-3′	173
	reverse: 5′-CCGGTGTTTTTGCAGAGTGG-3′	
*EGR1*	forward: 5′-GTTACCCCAGCCAAACCAC-3′	143
	reverse: 5′-TGGGTTGGTCATGCTCACT-3′	
*SLC25A4*	forward: 5′-GGGCTCTACCAGGGTTTCA-3′	150
	reverse: 5′-CGTCACACTCTGGGCAATC-3′	
*PDCD7*	forward: 5′-GCAGGAGGTGGAGGAGAAG-3′	178
	reverse: 5′-TGGAGGACAGACCCCTTTC-3′	
*MAPK11*	forward: 5′-TACCGGCAGGAGCTGAAC-3′	137
	reverse: 5′-TTCTTCACCGCCACCTTC-3′	
*MAPK12*	forward: 5′-CCACCTTCACCTTCCACCT-3′	91
	reverse: 5′-GCGTCTGCTCTGATGGATG-3′	
*FADD*	forward: 5′-CTGGGGAAGAAGACCTGTG-3′	150
	reverse: 5′-GCACACGCTCTGTCAGGTT-3′	
*BCL2*	forward: 5′-GGAGGATTGTGGCCTTCTTT-3′	176
	reverse: 5′-GCCGTACAGTTCCACAAAGG-3′	
*CASP2*	forward: 5′-TTGCCGAAGATGAGACTGC-3′	179
	reverse: 5′-GCGTTCACCTTAACCAGCA-3′	
*DAXX*	forward: 5′-AAGCCTCCTTGGATTCTGGT-3′	203
	reverse: 5′-ATCATCCTCCTGACCCTCCT-3′	
*FAS*	forward: 5′-AGTTGGGGAAGCTCTTTCACTT-3′	163
	reverse: 5′-CAGTCTTCCTCAATTCCAATCC-3′	
*REST*	forward: 5′-CGCCCATATAAATGTGAACTTTGTC-3′	145
	reverse: 5′-GGCGGGTTACTTCATGTTGATTAG-3′	
*GAPDH*	forward: 5′-GCACCGTCAAGGCTGAGAAC-3′	138
	reverse: 5′-TGGTGAAGACGCCAGTGGA-3′	
*BBC3* *(ChIP)*	forward: 5′-TTTCCGTCTGGGTGTGTGT-3′	153
	reverse: 5′-TCCAGGGTCCACAAAGTCA-3′	
*DAXX* *(ChIP)*	forward: 5′-CGCGTTGTGCTCATTTGT-3′	146
	reverse: 5′-TCCCATTTCCACGGCTTA-3′	
*SYN-1* *(ChIP)*	forward: 5′-GGTGCTGAAGCTGGCAGT-3′	169
	reverse: 5′-TGGGTTTTAGGACCAGGATG-3′	

## References

[1] Omuro A., DeAngelis L.M. (2013). Glioblastoma and other malignant gliomas a clinical review. J. Am. Med. Assoc..

[2] Grossman S.A., Ye X., Piantadosi S., Desideri S., Nabors L.B., Rosenfeld M., Fisher J., Consortium N.C. (2010). Survival of patients with newly diagnosed glioblastoma treated with radiation and temozolomide in research studies in the united states. Clin. Cancer Res..

[3] Wen P.Y., Kesari S. (2008). Malignant gliomas in adults. N. Engl. J. Med..

[4] Rockowitz S., Lien W.H., Pedrosa E., Wei G., Lin M., Zhao K., Lachman H.M., Fuchs E., Zheng D. (2014). Comparison of REST cistromes across human cell types reveals common and context-specific functions. PLoS Comput. Biol..

[5] Noh K.M., Hwang J.Y., Follenzi A., Athanasiadou R., Miyawaki T., Greally J.M., Bennett M.V., Zukin R.S. (2012). Repressor element-1 silencing transcription factor (REST)-dependent epigenetic remodeling is critical to ischemia-induced neuronal death. Proc. Natl. Acad. Sci. USA.

[6] Kellis M., Wold B., Snyder M.P., Bernstein B.E., Kundaje A., Marinov G.K., Ward L.D., Birney E., Crawford G.E., Dekker J. (2014). Defining functional DNA elements in the human genome. Proc. Natl. Acad. Sci. USA.

[7] Huang Z., Bao S. (2012). Ubiquitination and deubiquitination of REST and its roles in cancers. FEBS Lett..

[8] Zhao Y., Zhu M., Yu Y., Qiu L., Zhang Y., He L., Zhang J. (2016). Brain REST/NRSF is not only a silent repressor but also an active protector. Mol. Neurobiol..

[9] Ren H., Gao Z., Wu N., Zeng L., Tang X., Chen X., Liu Z., Zhang W., Wang L., Li Z. (2015). Expression of REST4 in human gliomas *in vivo* and influence of pioglitazone on REST *in vitro*. Biochem. Biophys. Res. Commun..

[10] Kamal M.M., Sathyan P., Singh S.K., Zinn P.O., Marisetty A.L., Liang S., Gumin J., El-Mesallamy H.O., Suki D., Colman H. (2012). Rest regulates oncogenic properties of glioblastoma stem cells. Stem Cells.

[11] Conti L., Crisafulli L., Caldera V., Tortoreto M., Brilli E., Conforti P., Zunino F., Magrassi L., Schiffer D., Cattaneo E. (2012). Rest controls self-renewal and tumorigenic competence of human glioblastoma cells. PLoS ONE.

[12] Zhang P., Lathia J.D., Flavahan W.A., Rich J.N., Mattson M.P. (2009). Squelching glioblastoma stem cells by targeting REST for proteasomal degradation. Trends Neurosci..

[13] Tivnan A., Zhao J., Johns T.G., Day B.W., Stringer B.W., Boyd A.W., Tiwari S., Giles K.M., Teo C., McDonald K.L. (2014). The tumor suppressor microRNA, miR-124a, is regulated by epigenetic silencing and by the transcriptional factor, REST in glioblastoma. Tumour Biol..

[14] Rosenbloom K.R., Sloan C.A., Malladi V.S., Dreszer T.R., Learned K., Kirkup V.M., Wong M.C., Maddren M., Fang R., Heitner S.G. (2013). ENCODE data in the UCSC genome browser: Year 5 update. Nucleic Acids Res..

[15] Szklarczyk D., Franceschini A., Wyder S., Forslund K., Heller D., Huerta-Cepas J., Simonovic M., Roth A., Santos A., Tsafou K.P. (2015). String v10: Protein–protein interaction networks, integrated over the tree of life. Nucleic Acids Res..

[16] Meyer M., Reimand J., Lan X., Head R., Zhu X., Kushida M., Bayani J., Pressey J.C., Lionel A.C., Clarke I.D. (2015). Single cell-derived clonal analysis of human glioblastoma links functional and genomic heterogeneity. Proc. Natl. Acad. Sci. USA.

[17] Cancer Genome Atlas Research Network (2008). Comprehensive genomic characterization defines human glioblastoma genes and core pathways. Nature.

[18] Jackson A.L., Linsley P.S. (2010). Recognizing and avoiding siRNA off-target effects for target identification and therapeutic application. Nat. Rev. Drug Discov..

[19] Das C.M., Taylor P., Gireud M., Singh A., Lee D., Fuller G., Ji L., Fangusaro J., Rajaram V., Goldman S. (2013). The deubiquitylase USP37 links REST to the control of p27 stability and cell proliferation. Oncogene.

[20] Dietrich N., Lerdrup M., Landt E., Agrawal-Singh S., Bak M., Tommerup N., Rappsilber J., Sodersten E., Hansen K. (2012). REST-mediated recruitment of Polycomb repressor complexes in mammalian cells. PLoS Genet..

[21] Galderisi U., Jori F.P., Giordano A. (2003). Cell cycle regulation and neural differentiation. Oncogene.

[22] Wang X., McGowan C.H., Zhao M., He L., Downey J.S., Fearns C., Wang Y., Huang S., Han J. (2000). Involvement of the MKK6-p38γ cascade in γ-radiation-induced cell cycle arrest. Mol. Cell. Biol..

[23] Liu R., Tian B., Gearing M., Hunter S., Ye K., Mao Z. (2008). Cdk5-mediated regulation of the PIKE-A-Akt pathway and glioblastoma cell invasion. Proc. Natl. Acad. Sci. USA.

[24] Calogero A., Lombari V., de Gregorio G., Porcellini A., Ucci S., Arcella A., Caruso R., Gagliardi F.M., Gulino A., Lanzetta G. (2004). Inhibition of cell growth by EGR-1 in human primary cultures from malignant glioma. Cancer Cell Int..

[25] Yeo M., Lee S.K., Lee B., Ruiz E.C., Pfaff S.L., Gill G.N. (2005). Small CTD phosphatases function in silencing neuronal gene expression. Science.

[26] Roopra A., Qazi R., Schoenike B., Daley T.J., Morrison J.F. (2004). Localized domains of G9a-mediated histone methylation are required for silencing of neuronal genes. Mol. Cell.

[27] Shi Y.J., Matson C., Lan F., Iwase S., Baba T., Shi Y. (2005). Regulation of LSD1 histone demethylase activity by its associated factors. Mol. Cell.

[28] Riccio A. (2010). Dynamic epigenetic regulation in neurons: Enzymes, stimuli and signaling pathways. Nat. Neurosci..

[29] Jaenisch R., Bird A. (2003). Epigenetic regulation of gene expression: How the genome integrates intrinsic and environmental signals. Nat. Genet..

[30] Zhang D., Wang J., Wang Z., Zhang T., Shi P., Wang X., Zhao F., Liu X., Lin X., Pang X. (2015). miR-136 modulates TGF-β1-induced proliferation arrest by targeting PPP2R2A in keratinocytes. BioMed Res. Int..

[31] Barrachina M., Moreno J., Juves S., Moreno D., Olive M., Ferrer I. (2007). Target genes of neuron-restrictive silencer factor are abnormally up-regulated in human myotilinopathy. Am. J. Pathol..

[32] Lu T., Aron L., Zullo J., Pan Y., Kim H., Chen Y., Yang T.H., Kim H.M., Drake D., Liu X.S. (2014). REST and stress resistance in ageing and Alzheimer’s disease. Nature.

